# Plants *in vitro* propagation with its applications in food, pharmaceuticals and cosmetic industries; current scenario and future approaches

**DOI:** 10.3389/fpls.2022.1009395

**Published:** 2022-10-13

**Authors:** Ammarah Hasnain, Syed Atif Hasan Naqvi, Syeda Iqra Ayesha, Fatima Khalid, Manahil Ellahi, Shehzad Iqbal, Muhammad Zeeshan Hassan, Aqleem Abbas, Robert Adamski, Dorota Markowska, Alaa Baazeem, Ghulam Mustafa, Mahmoud Moustafa, Mohamed E. Hasan, Mohamed M. A. Abdelhamid

**Affiliations:** ^1^ Institute of Molecular Biology and Biotechnology, The University of Lahore, Lahore, Pakistan; ^2^ Department of Plant Pathology, Faculty of Agricultural Sciences and Technology (FAST), Bahauddin Zakariya University, Multan, Pakistan; ^3^ College of Plant Sciences and Technology, Huazhong Agricultural University, Wuhan, China; ^4^ State Key Laboratory of Agricultural Microbiology and Provincial Key Lab of Plant Pathology, Huazhong Agricultural University, Wuhan, China; ^5^ Faculty of Process and Environmental Engineering, Lodz University of Technology, Lodz, Poland; ^6^ Department of Biology, College of Science, Taif University, Taif, Saudi Arabia; ^7^ Department of Agriculture (Extension and Adoptive Research), Agriculture Extension Department of Government of Punjab, Lahore, Pakistan; ^8^ Department of Biology, Faculty of Science, King Khalid University, Abha, Saudi Arabia; ^9^ Department of Botany and Microbiology, Faculty of Science, South Valley University, Qena, Egypt; ^10^ Bioinformatics Department, Genetic Engineering and Biotechnology Research Institute, University of Sadat City, Sadat City, Egypt; ^11^ Agricultural Botany Department, Faculty of Agriculture (Saba Basha), Alexandria University, Alexandria, Egypt

**Keywords:** plant tissue culture, explants, secondary metabolites, industry, pharmaceuticals, medicines, cosmetics

## Abstract

Plant tissue culture technique employed for the identification and isolation of bioactive phytocompounds has numerous industrial applications. It provides potential benefits for different industries which include food, pharmaceutical and cosmetics. Various agronomic crops i.e., cereals, fruits, vegetables, ornamental plants and forest trees are currently being used for *in vitro* propagation. Plant tissue culture coupled with biotechnological approaches leads towards sustainable agricultural development providing solutions to major food security issues. Plants are the rich source of phytochemicals with medicinal properties rendering them useful for the industrial production of pharmaceuticals and nutraceuticals. Furthermore, there are numerous plant compounds with application in the cosmetics industry. In addition to having moisturizing, anti‐ageing, anti‐wrinkle effects; plant-derived compounds also possess pharmacological properties such as antiviral, antimicrobial, antifungal, anticancer, antioxidant, anti-inflammatory, and anti-allergy characteristics. The *in vitro* propagation of industrially significant flora is gaining attention because of its several advantages over conventional plant propagation methods. One of the major advantages of this technique is the quick availability of food throughout the year, irrespective of the growing season, thus opening new opportunities to the producers and farmers. The sterile or endangered flora can also be conserved by plant micro propagation methods. Hence, plant tissue culture is an extremely efficient and cost-effective technique for biosynthetic studies and bio-production, biotransformation, or bioconversion of plant-derived compounds. However, there are certain limitations of *in-vitro* plant regeneration system including difficulties with continuous operation, product removal, and aseptic conditions. For sustainable industrial applications of *in-vitro* regenerated plants on a large scale, these constraints need to be addressed in future studies.

## 1 Introduction

Plant tissue culture is a technique in which fragments of tissues from a plant (explants) are developed *in vitro* in an artificial medium under aseptic conditions. It involves culturing explants (such as shoot tip, root tip, callus, seed, embryo, pollen grain, ovule or even a single cell) isolated from mother plant on asterile nutrient medium which leads to cell multiplication and plant regeneration (Ravishankar and Venkataraman, 1990; Veeresham and Chitti, 2013; Kaya and Huyop, 2020; Vidyagina et al., 2021). The commonly used medium in plant tissue culture is Murashigeand Skoog (MS) basal medium (Murashige and Skoog, 1962) supplemented with the required amounts of plant hormones which include auxins, cytokinins, abscisic acid, gibberellins, ethylene, and growth regulators with similar metabolic effects (Gaspar et al., 1996; Arab et al., 2014; FargosoMonfort et al., 2018; Kaya and Karakütük, 2018).

The first reports on tissue culture came from the early twentieth century when Gottlieb Haberlandt (Haberlandt, 1902) undertook experiments to sustain mesophyll cells in culture based on postulates establishing the “toti potentiality of plant cells.” Since then, progress has been steady, with hundreds of results and publications on the use of tissue culture techniques in breeding programmes, genetic biodiversity conservation, and biopharmaceutical manufacture being reported each year (García-Gonzáles et al., 2010). Initially, plant tissue culture was utilized as a research tool to study the development of isolated fragments of plant cells and tissues; however, the advent of recent molecular biology techniques significantly broadened the avenue of experimental investigations and applications (Chen et al., 2016; Tegen and Mohammed, 2016).

**Figure 1:**
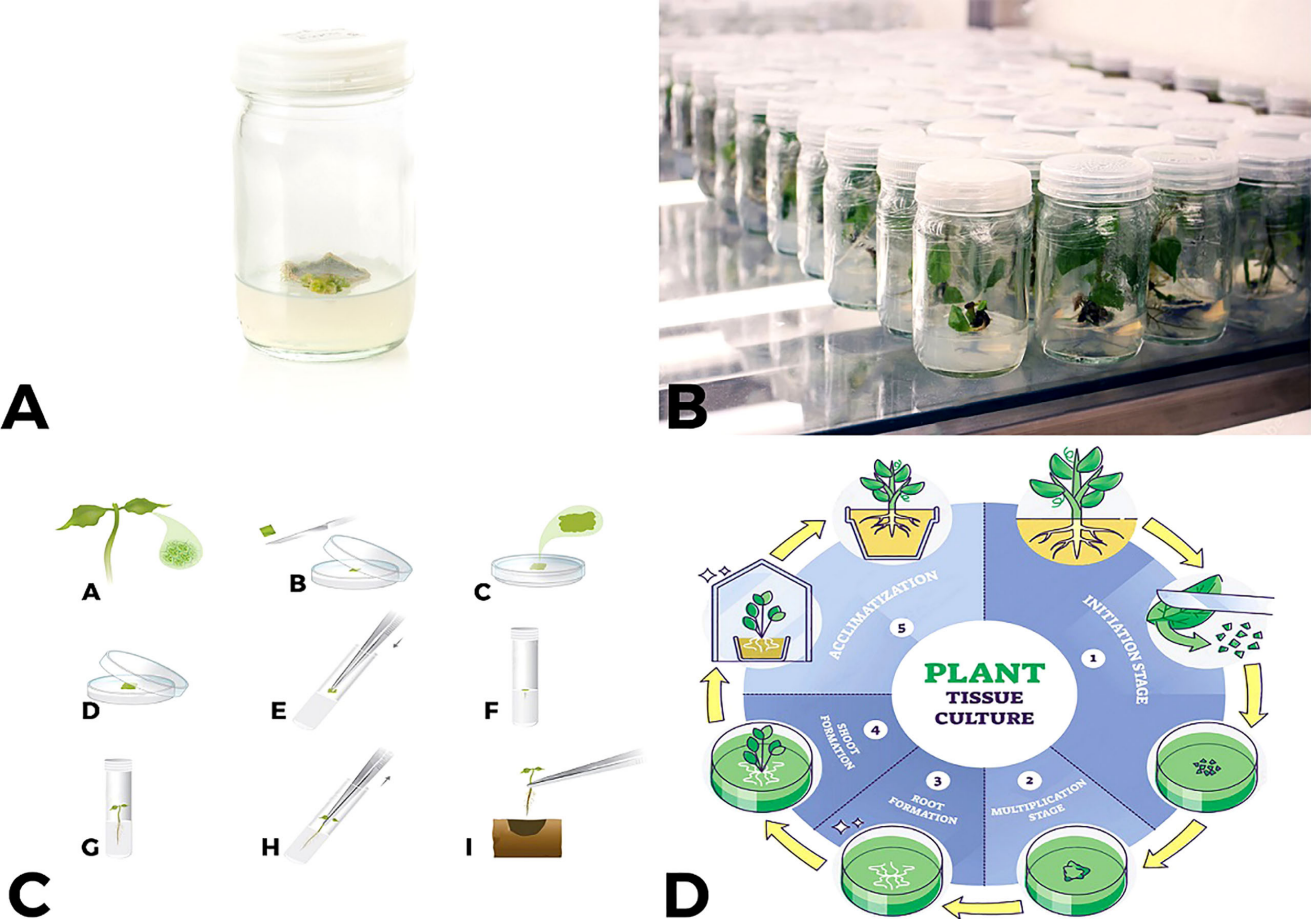
An overview of tissue culture process **(A, B)** small explant develops callus which then produces shoots a few weeks after being placed into tissue culture media **(C)** “A to I” shows complete procedure from single cell placement to MS media to development of a complete plant **(D)** How all phases in plant tissue culture from initiation, multiplication, root formation, shoot formation and acclimatization occurs.

**Figure 2:**
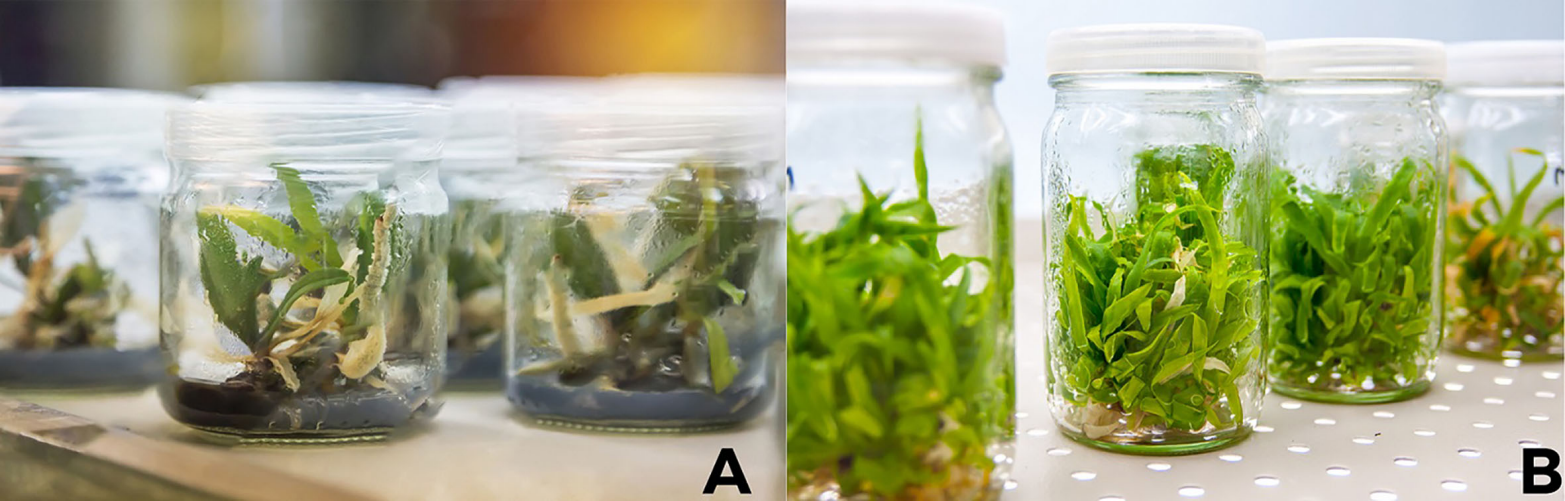
*In vitro* plant propagation of plants at Tissue Culture Lab **(A, B)** Roots are fully developed prior to moving plants to pots of soil.

Plant tissue culture plays a significant role in basic research in the areas of plant pathology, plant physiology, plant metabolites and conservation (Espinosa-Leal et al., 2015; Chandran et al., 2020; Vidyagina et al., 2021; Taalat et al., 2021). Plant micro-propagation has extensively been used to have an insight into plant pathology studies which include factors influencing penetration, infection and multiplication of pathogens, the nature of irregular cell division or growth, and the morphogenetic potential of the diseased cell (Maheshwari, 1969; Köhl et al., 2019). The employment of plant tissue culture in plant pathology is not only restricted to use plant tissues as a substrate for the pathogens, but it provides the basic understanding of various characteristics of pathological growth, pathogens’ attack weapons, and the host response to an infection caused by the invading organism (Maheshwari, 1969). Plant physiology and plant morphogenesis requires the capability to grow plants *in vitro* that might be best accomplished with plant tissue culture procedures (Pullaiah and Subba, 2009). Furthermore, the conservation of plant biodiversity is indispensable for future crops safety due to increasing challenges of biotic and abiotic stresses (Alexandratos and Bruinsma, 2012; Gupta et al., 2016). In this regard, *in vitro* techniques permit improvement in various traits associated with plant growth and yield that can, later, be used for ex-situ conservation (Lavanya et al., 2014). The active plant compounds obtained from rare or endangered species can be manufactured by *in vitro* techniques without adverse environmental effects and in agreement with the bio- sustainability matters that the market demands (**Figures 1**, **2**).

The *in vitro* plant propagation has not only made a significant contribution in the knowledge of basic research, but it also offers potential applications as it guarantees a sustainable industry that relies on commercial production of plant-derived compounds (Kumari et al., 2021; Chandran et al., 2020). The culture of plant tissues is an effective instrument for the isolation and processing of active compounds, including secondary substances and engineered molecules, from economically important plants (Espinosa-Leal et al., 2018; Kumari et al., 2021). Due to advancement in contemporary techniques, several protocols have been developed for the production of a wide variety of plants secondary metabolites on a commercial scale (Shasmita and Naik, 2018). Furthermore, plant tissue culture coupled with biotechnological approaches is applicable to the development of genetically modified plants as well as embryo rescue procedures (Altpeter et al., 2016; Tegen and Mohammed, 2016; Sadiku et al., 2018). It plays a pivotal role in vector-mediated or vector-independent gene-delivery into plant genome for the production of transgenic plants with improved traits (Hussain et al., 2012; Gleba et al., 2014; Hasnain et al., 2020).

Various crops with superior traits have been developed using this technology with enhanced nutritional value and biotic/abiotic stress resistance that leads to increased crop yield (Ravishankar and Venkataraman, 1990; Rishirumuhirwa, 2007; Ragavendran and Natarajan, 2017). Different transcription factors which regulate nutrient assimilation pathways have been over expressed in staple crops that may improve crop yield (Kurai et al., 2011; Hasnain et al., 2020). Plant propagation using tissue culture techniques have also provides a valuable commercial prospect in the industrial manufacture of ornamental plants, vegetable and fruit plants with economically important products (Hussain et al., 2012). The current review gives insights into the research that has been conducted successfully on *in-vitro* plant regeneration in order to obtain valuable bioactive phytocompounds having several applications. The review also focuses on major constraints associated with the use of plant tissue culture technique that need to be addressed for sustainable industrial applications. Various useful products can be obtained as food products, pharmaceutical products and cosmetics *via in vitro* plant regeneration (Niazian et al., 2017; Chandran et al., 2020). However, plant tissue culture studies are needed to be conducted on a large scale in order to ensure food security and meet the demand of food supply of increasing human population (Ragavendran and Natarajan, 2017). In this review we have discussed micro-propagation of some plants species to have a better understanding of significance of pant micro-propagation in different industries.

## 2 Plant propagation types

The two types of plant propagation are mentioned as follows

### 2.1 Macro-propagation

Macro-propagation is a method used in a shed or in the field. It entails producing suckers from clean planting material by eliminating apical dominance. Macro-propagation techniques are divided into two types: field-based techniques that rely on total or partial decapitation, and detached corm techniques that are done in a shed. Traditional methods of offset planting, rhizome planting, cutting roots, and layering are used in macro-propagation (Njukwe et al., 2007).

### 2.2 Micro-propogation

Plant micro propagation, also known as plant tissue culture, is a technique that isolates, sterilizes, and incubates cells, tissues, or organs of chosen plants in a growth-promoting aseptic environment to create a large number of plantlets. The isolated cloning technique revealed that, given the right conditions, somatic cells may develop into a complete plant (Njukwe et al., 2007).

## 3 Plant tissue culture methods

Several methods are available for plant tissue culture. In organogenesis, the commonly used method,organ formation can occur directly from meristems, or indirectly from dedifferentiated cells (callus). The resultant cultures can then be utilized to mass produce plants (micro propagation) or to develop specific organs (e.g., roots in hairy root culture) (Espinosa-Leal et al., 2018).

### 3.1 Organogenesis

Organogenesis is the production of plant organs from a specific tissue in order to develop complete plants. It is characterized by being polar, which means that just one aerial organ or root is released and a new complete plant is generated from this. Simultaneously, organogenesis can be direct in which the organogenic shoot is produced directly from the explants, or indirect, in which the organogenic process happens from previously created callus in the original explants (Gupta et al., 2020).

### 3.2 Somatic embryogenesis

Somatic embryogenesis is the process of producing embryos from somatic plant cells (any non-sexual cell) in order to produce a whole plant. In contrast to organogenesis, this is a polar process in which the aerial structures and roots of plants develop from the somatic embryo. It can either be direct or indirect, depending on whether the process begins with the original explants or with previously produced callus (Gupta et al., 2020).

## 4 Types of tissue culture

### 4.1 Callus culture

Callus is an undifferentiated mass of tissue that forms on explants after a few weeks on growth medium with appropriate hormones. Callus development is the result of a well-known process known as de-differentiation or re-differentiation. To stimulate callus induction and development, several growth hormones are employed. Callus induction and development were increased in *Cephaelis ipecacuanha* by 2,4-D, NAA, and kinetin. Organogenesis allows for the effective regeneration of new plants from callus (Ujjwala, 2007; Espinosa-Leal et al., 2018).

### 4.2 Suspension culture

*In vitro* suspension cultures are created when friable calli are grown on liquid medium in a suitable container and regularly agitated to provide free cell suspension. Conical flasks are utilized because of its enormous surface area, which aids in the retention of liquid medium and the constant exchange of gases. Suspension cultures are classified as batch or continuous cultures. At regular intervals, a part of the original cell suspension is collected and sub-cultured on to fresh medium in batch cultures. In continuous cultures, new media is introduced to the same culture on regular basis, and surplus cell suspensions are discarded. Suspension cultures are commonly utilized in large-scale synthesis of secondary metabolites. Chemostat bioreactors are the devices particularly built for large-scale continuous culturing (Smith and Drew, 1990a; Smith and Drew, 1990b; Baezas-Lopez, 1995; Kodym and Zapata-Arias, 2001; Ahloowalia et al., 2004; Bimonte et al., 2011; Sidhu, 2011; Chen et al., 2015; Chen et al., 2016a; Titos et al., 2016; Suman, 2017).

### 4.3 Meristem culture

The culture in which tiny excised shoot apices are cultivated, each having an apical meristemetic dome with one or two leaf primordia. Typically, the shoot apex is developed to produce a single shoot (Ahluwalia et al., 2016).

### 4.4 Protoplast culture

Protoplasts are plant cells with cell walls removed by enzymatic or mechanical methods. Protoplasts are obtained by immersing plant cells in a hypertonic solution, which causes the plasma membrane to shrink off the cell walldue to water efflux. Cell wall may now be removed using either enzymatic digestion with pectinase and cellulose, or by mechanical techniques (Sidhu, 2011).

### 4.5 Shoot tip culture

Shoot tip culture is developed from excised shoot tips/buds larger than the shoot apices (used for meristems cultures), and had several leaf primordia. These shoot apices are often cultivated such that each one generates several shoots (Espinosa-Leal et al., 2018).

### 4.6 Lateral bud node culture

Lateral bud node culture is carried out on a short piece of stem tissue where stem portions carrying single or many nodes may be cultivated. Each bud is cultivated to produce a single shoot (Sidhu, 2011).

### 4.7 Isolated root culture

In isolated root culture, a branching root system can be generated by growing roots that are not attached to shoots (Ahluwalia et al., 2016).

### 4.8 Embryo culture

Embryo culture has fertilized or unfertilized zygotic (seed) embryos dissected from maturing seeds or fruits and cultivated *in vitro* until seedling formation. Embryo culture is not the same as somatic embryogenesis (Gupta et al., 2020).

## 5 Advantages of *in vitro* plant propagation

Plant micro-propagation with different explants like seeds, embryos, calli, anthers, protoplasts, and meristemetic tissues of root/shoot tips is used for large-scale production of industrial products (Mali and Chavan, 2016; Shasmita and Naik, 2018). Conventionally, somatic hybridization, which produces interspecific and intergeneric hybrids, was commonly used as an essential method for plant breeding. The procedure entails the fusion of two somatic protoplasts followed by the selection of desirable hybrid cells and then regeneration of hybrid plants (Lambardi et al., 2008). Protoplast fusion is an effective method of transferring genes with desirable traits from one species to another with a great impact on crop development. Somatic hybrids created from rice and ditch reed by electrofusion exhibited better results against salt stress (Hussain et al., 2012). The most recent feature of plant cell and tissue culture is genetic transformation, which allows for the transfer of genes with desirable traits into host plants and the recovery of transgenic plants (Hinchee et al., 1994). This approach offers a high potential for the development of agricultural plants with novel traits. The genetically modified plants exhibit agronomically significant features such as greater yield, improved nutritional quality, improved pest and disease resistance (Sinclair et al., 2004).

Recent breakthroughs in plant cell culture, molecular biology, enzymology, and fermentation technology indicate that these systems are a viable source of synthesis ofimportant secondary metabolites. Plants infected with an engineered virus generate relatively significant amounts of desired chemicals, and these plants can sustain steady levels of protein synthesis without extra intervention (Sajc et al., 2000). Large-scale plant tissue culture has been shown to be an appealing alternative approach to traditional plantation methods since it provides a regulated supply of biochemicals independent of plant availability (Sajc et al., 2000). Kieran et al. (1997) examined the effects of several engineering parameters on cell suspension cultures for the recovery of important metabolites (Vanisree et al., 2004). Tissue culture technology advancements show that transcription factors are effective new molecular tools for plant metabolic engineering to boost the synthesis of important chemicals (Gantet and Memelink, 2002; Hasnain et al., 2020). *In vitro* cell culture has the inherent advantage of producing therapeutic proteins such as monoclonal antibodies, antigenic proteins that act as immunogens, human serum albumin, interferon, immuno-contraceptive proteins, antihypertensive drug angiotensins, and human haemoglobin in certain situations (Wongsamuth and Doran, 1997).

Tissue culture technique offers several advantages over plant propagation under natural conditions (Bhojwani and Dantu, 2013; Chandran et al., 2020). It is a rapid procedure as thousands of seedlings can be produced from small fragments of plants in a short period of time in contrast to conventionally propagated flora (Mattick, 2018). This also helps to accelerate the production process of new crop varieties with superior traits as tissue culture experiments require less time and space compared to *in-vivo* plant growth (Krasteva et al., 2020). Tissue culture can be used to propagate perennial plants; irrespective of weather or season (Ahmad and Anis, 2007). It also helps in the development of pathogen-free micro-plants saved from various diseases and the new plants produced by tissue culture under aseptic conditions are also sterile (Chiipanthenga et al., 2012; Bayoudh et al., 2015; Tegen and Mohammed, 2016). Furthermore, several plant species generate resistant seeds that cannot be retained for extended periods of time; in this situation, tissue culture can be utilized for plant conservation in vegetative state, generally under slow growth conditions (Lambardi et al., 2008), or for cryopreservation (García-Gonzáles et al., 2010). In some cases, inter-specific and inter-generic hybrids can be obtained using embryo rescue technique which is not possible through conventional methods (Mattick, 2018). Tissue culture has been extensively utilized in breeding programs for over 50 years. The hybrids of such crosses are often sterile due to embryo abortion but can be ‘rescued’ by means of culturing or transplanting the embryos. The media conditions used for the cell culture can be modified accordingly to provide more acceptable results for the performed experiments (Pant, 2014).

## 6 Applications of plant tissue culture

Since earliest times, humanity has been dependent on plants for food, flavors, medicines and many other uses (Rai et al., 2011). Because the spectrum of phytochemicals is larger than that of any other class of creature, plants are the most plentiful source of herbal remedies in the food and cosmetics sectors (Barbulova et al., 2014). Plant tissue culture can be used for a wide range of purposes with various applications in research and industry (Rai et al., 2011; Niazian et al., 2017; Chandran et al., 2020). The major commercial applications which *in vitro* plant propagation offers are in the following major industries (**Table 1**).

**Figure 3:**
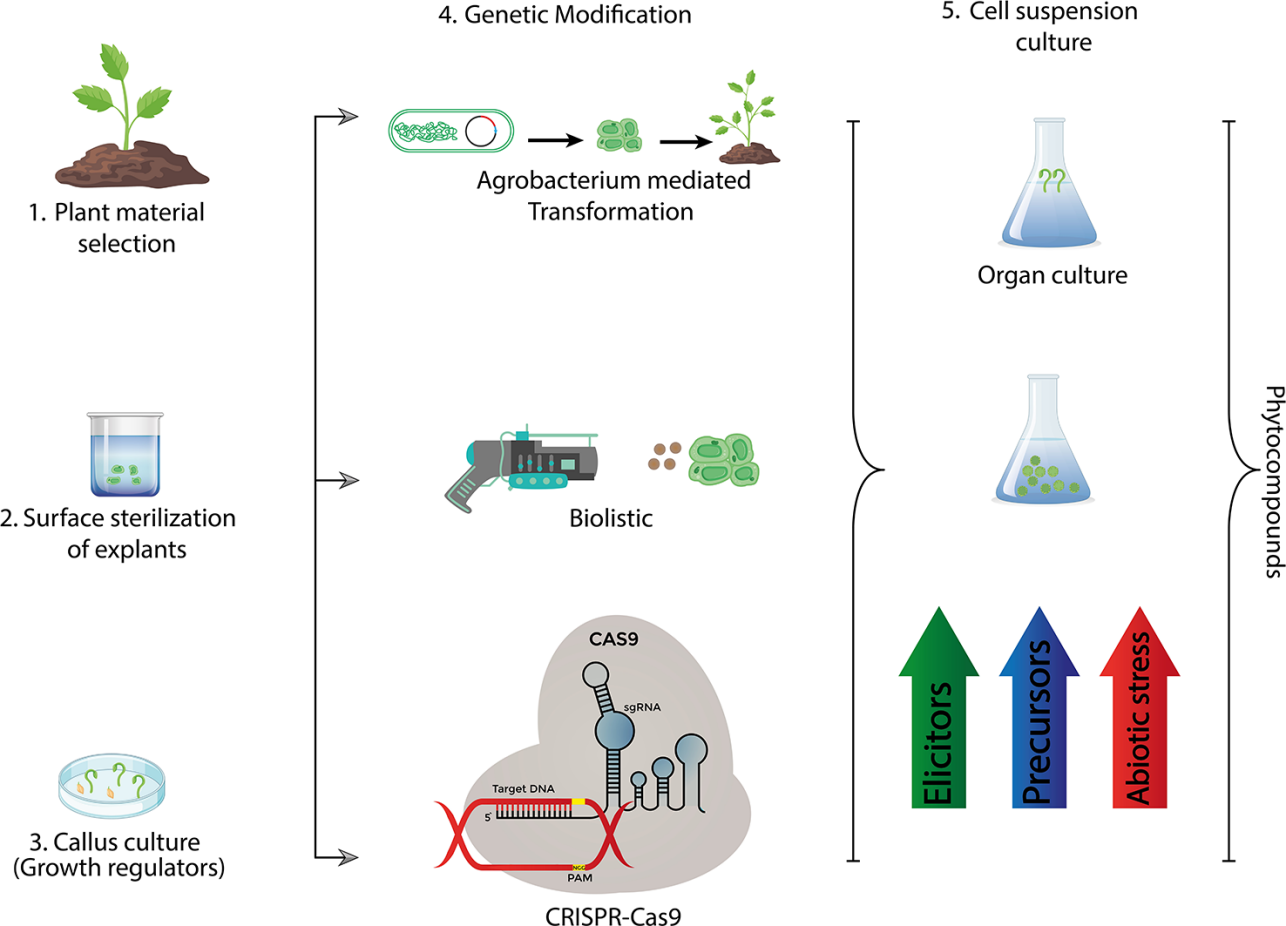
Recent methods used for industrial production of bioactive compounds *via* plant tissue culture.

**Table 1:** Applications of plant tissue culture technique in different industries.

Industry	Products	Applications and advantages	Limitations	References
Food	Rice	Gives high yield irrespective of seasonal requirements and require less area for production	Produce less resilient plants, Less acceptance towards tissue cultured food plants in general public, no need of herbicide and pesticide application	(Roy et al., 2000; Sparks and Jones, 2014; Yaqoob et al., 2016; Hasnain et al., 2018; Hasnain et al., 2020)
	Wheat	Wheat varieties are resistant against different diseases with increased yield and high nutritional value		
	Pineapple (Smooth Cayenne)	High yield, Gives efficient and fast production of required flora		
	Banana	Cheap and sterile planting materials ultimately giving a high yield during whole year		
	Chocolate	Unique taste and aroma		
Pharmaceutical	Codeine	To relieve pain as well as coughing	Less production of secondary metabolites, difficult to remove secondary metabolites from the culture	(Czyz et al., 2014; Hayden et al., 2014; Mali and Chavan, 2016)
	Atropine	Treat the symptoms of decreased heart rate		
	Reserpine	Treat high blood pressure		
	Hyoscyamine	Treat a range of gastrointestinal disorders		
	Digoxin	Used to treat heart failure		
	Scopolamine	Avoid nausea and vomiting induced by motion sickness		
	Morphine	Pain killer		
Cosmetics	Rubusidaeushydrosoluble extract	Anti-inflammatory activity in skin cells	It is difficult to maintain aseptic conditions resulting high cost.	(Ebile et al.,2018)
	Nicotianasylvestriscell wall preparation	Collagen synthesis and protection in skin cells		
	Coffeabengalensishydrosoluble extract	Epidermal hydration and collagen synthesis in skin cells		
	Dolichosbiflorushydrosoluble extract	Anti-inflammatory activity and UV damage protection		
	Malusdomesticus whole lysate			
		Reversion of aging signs		

### 6.1 Food industry

Plants are main source of life on planet earth for living creatures and exclusively providing nutrition to humans and being the source of phyto-constituents like starches, proteins, dietary fibers, minerals and cancer preventive agents (Khosroshahi et al., 2006; Nasr et al., 2007). Plant tissue culture is a powerful tool of agricultural improvement and offers tangible solutions to major crop problems that arise due to constant threat of biotic and abiotic stresses minimizing the crop yield (Ragavendran and Natarajan, 2017; Eibl et al., 2018). Plant tissue culture coupled with different biotechnological approaches leads to a sustainable agricultural ensuring food productivity and safety (Alexandratos and Bruinsma, 2012; Bhojwani and Dantu, 2013). Various transgenic plants including Arabidopsis, wheat and tobacco are developed through genetic engineering and plant tissue culture conferring resistance against different environmental stresses (Voytas and Gao, 2014; Hasnain et al., 2020). Since plant tissue culture is simple, low-cost and environment friendly, it is imperative to employ this technique for the development of sustainable agriculture in order to meet the food demand of increasing human population (Gahakwa et al., 2012; Hussain et al., 2012; Altpeter et al., 2016). The greatest value of plant cell/tissue culture rests not so much on their application to mass clonal propagation (micro propagation), but also in their involvement in plant improvement and bio-processing. Its uses extend well beyond crop cultivation and productionas it has an enormous potential of bridging the gap between the research institutes and industry. The agro-industry based projects are crucial to achieve agricultural sustainability enabling the ultimate target, the stakeholder, reap benefit of extensive research being done across the globe (Suman, 2017). In this review we have discussed micro-propagation of some fruits and crops with high nutritional value to have a better understanding of role of plant tissue culture towards sustainable agriculture (**Figure 3**).

#### 6.1.1 Banana tissue culture

Common bananas are cultivars of *Musa acuminate*, native to South Asia. They are one of the essential fruits that are being consumed as a staple food in the developing countries (Njuguna et al., 2003; Dubois et al., 2013). Banana is also regarded as an important export produce in tropic regions of the world; however, its production has been reduced during the last few years (Macharia et al., 2010; Liu et al., 2019). Because banana is an essential food crop and the second most significant fruit crop after mango, considerable care has been given to recording the stages needed in effective micro propagation of banana. Despite the banana crop’s high nutritional and economic value, the major production restriction is due to lack of dependable and safe planting material. Diseases, pests, and a lack of planting materials have all been blamed for the drop in production. Planting materials obtained by traditional means (suckers) are insufficient to fulfill the growing demand for planting and are of low quality. Tissue culturing is an efficient method for addressing problems pertaining to its low production (Strosse et al., 2004). The challenge involved with conventional banana breeding is better understood by the fact that no new commercially suitable banana cultivar has been generated over 60 years of continuous breeding efforts (Rowe, 1984). In fact, bananas are one of the few crops that are grown using exclusively clones produced from natural somatic mutations.Each mother plant produces one to eight suckers in line, which is insufficient (Ko et al., 2009; Rossmann et al., 2012). The solution is to propagate cheap and sterile planting materials using tissue culture technique whilethe micro propagation of bananas will not only lead to fast multiplication of desirable banana cultivars butproduces disease-free planting material may also be made available throughout the year (Dubois et al., 2013; Liu et al., 2019; Selvakumar and Parasurama, 2020).

Agricultural diversification to satisfy our future demands necessitates the implementation of innovative agricultural technology. The finest cultural methods, excessive fertilizers, and pest control procedures will not yield the desired results unless the best planting material is used. Tissue culture is now widely being used as a viable horticultural propagation technology, and it has changed the horticultural business. This approach is used to achieve mass proliferation and the creation of disease-free stock material (Suman, 2017).

**Figure 4:**
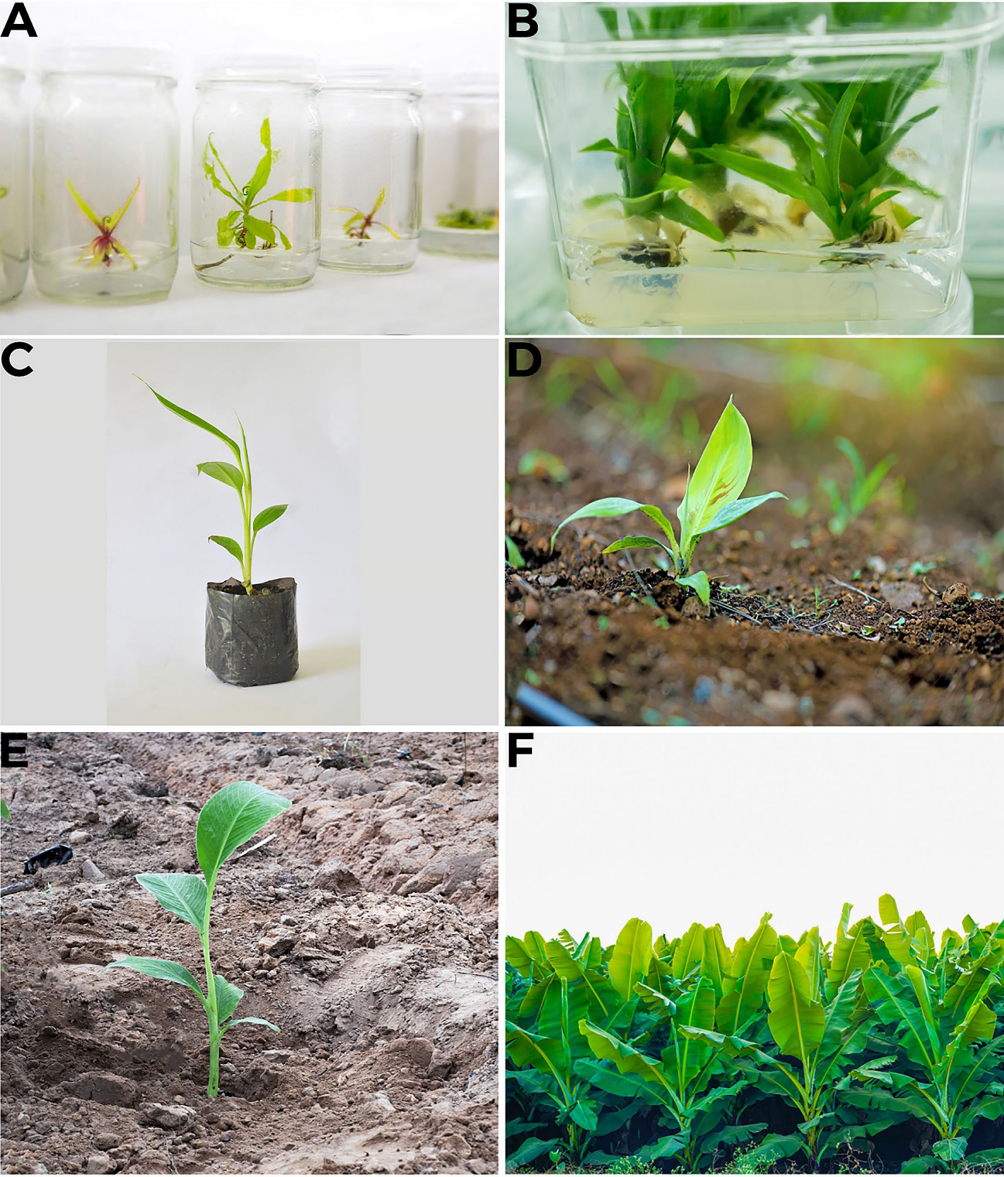
*In vitro* propagation of banana **(A)** Callus formation to roots development **(B)** Maturation of plants in media **(C)** Plant sown in pot **(D)** Plant sown in soil in controlled conditions, acclimatization **(E)** Sowing of plant in the field **(F)** Tissue culture produced Banana field.

Various studies depict that micro propagated bananas are capable of outperforming compared to conventional planting material techniques (Smith and Drew, 1990a; Dubois et al., 2013; Suman, 2017). However, the prevalence of somaclonal variations, referred to as off-types in the industry, remained the most serious difficulty that developed to limit widespread acceptance of micropropagated banana planting material. Plantings from commercial laboratories have been found to include up to 90% off-types (Walduck et al., 1988), with dwarfism being the most prevalent off-type detected. Dwarfs are more prone to ‘choke-throat,’ a physiological condition in which the bunch fails to emerge entirely from the plant, and their hands are more densely packed, making dehanding more difficult (Smith, 1988).These difficulties can be overcome by altering the type and concentrations of phytohormones in culture media, the number of subcultures, and selecting different types of explantse.g. axillary buds are more stable than adventitious buds. One of the most important intrinsic determinants is the genetic stability of the cultivar or genotype. While there is little that can be done to control intrinsic variables, micro propagation, is comparatively useful in modulating the intrinsic factors by controlling external factors that would be, otherwise, difficult under natural conditions (Walduck et al., 1988; Hasnain et al., 2020). Commercial laboratories are now using tissue culture protocols that minimize somaclonal variations, and working on creating accurate screening and selection approaches for early detection of off-types.Although somaclonal variation is deleterious to quick clonal multiplication, some off-types have been discovered that have significant agronomic utility. ‘Mons Mari,’ a micropropagated Cavendish cultivar, with extra-long finger length for all hands and fruit (2-3 cm) longer than typicalis one of the examples. This selection has the potential to increase profits through extra-large fruit sales. A dwarf form with no visible choke-throat difficulties, larger, more open hands, and may exhibit higher wind resistance than ‘Williams’ isanother option. These plants are now being replicated for field testing in a variety of Queensland locations (Walduck et al., 1988; Bestoso et al., 2006; Chen et al., 2016b; Reinhardt et al., 2018) (**Figure 4**).

#### 6.1.2 Pineapple tissue culture

**Figure 5:**
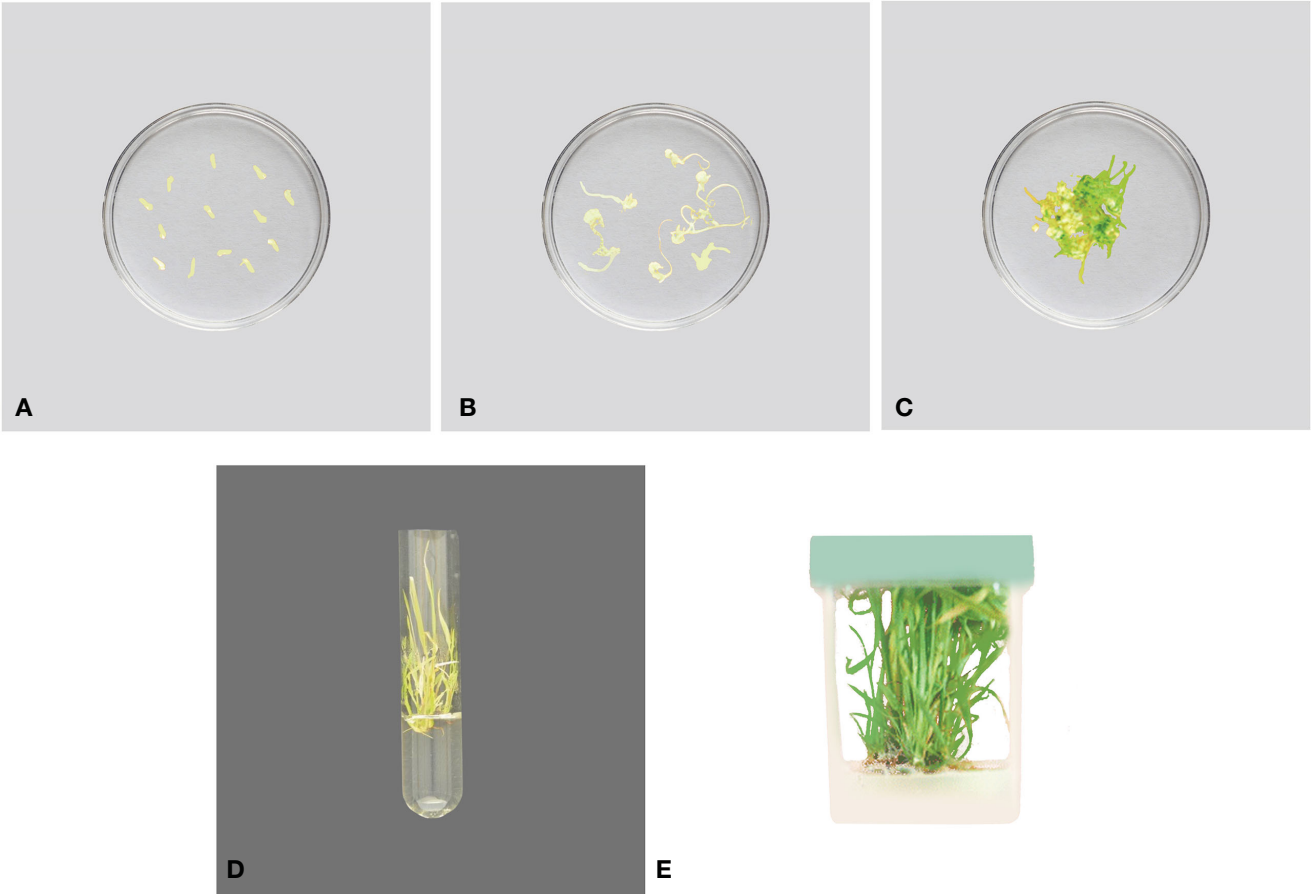
*In vitro* propagation of pineapple **(A, B)** Initiation and multiplication stage **(C–E)** Root and shoot formation, plants are fully grown to move to pots of soil.

In tropical places, the pineapple is a popular vegetatively propagated crop; nevertheless, pineapple production is limited due to a lack of planting materials (Be and Debergh, 2006; Nikumbhe et al., 2013). Suckers and slips were traditionally used to propagate pineapple, resulting in a limited number of seedlings that did not meet farmer demand. Tissue culture provides a practical technique to produce the needed amount of desired flora in this regard (Roy et al., 2000). Explants produced from crown-tip meristems are sterilized and cultivated on MS media enriched with cytokinins in an efficient and cost-effective procedure for commercial micro-propagation of Smooth Cayenne (a spine-free variety of pineapple). Direct organogenesis is used to regenerate several shoots, which are then rooted in auxins-supplemented medium. After that, the rooted shoots are planted in a sterilized soil substrate (Usman et al., 2013; Akin-Idowu et al., 2014; Reinhardt et al., 2018). In other cases, auxiliary buds are taken from slips or suckers and put on different medium to promote explant establishment followed by single shoot growth, shoot proliferation, and finally root initiation. Preliminary studies on*in vitro* propagation of pineapple by Geoge and Sherrington (1984) demonstrated that multiplication rates of 30 to 50 per month were obtained on medium containing benzylaminopurine (BAP), compared to 4-5 per year with traditional propagation. The use of large quantities of cytokinins in multiplication media, on the other hand, has been linked to the induction of somaclonal variations (Geoge and Sherrington, 1984). Plantings from commercial laboratories with a multiplication rate of four per month contained a maximum of 5% variations (predominantly rough-leaved types) from a total of ten thousand plants. This indicates an appropriate amountgiven the benefits of nursery establishment.Moreover, pineapple plants in their first generation of *in vitro* propagation have shown a high number of slips and suckers. This has been beneficial for increasing planting material in nursery blocks (**Figure 5**).

#### 6.1.3 Wheat tissue culture

**Figure 6:**
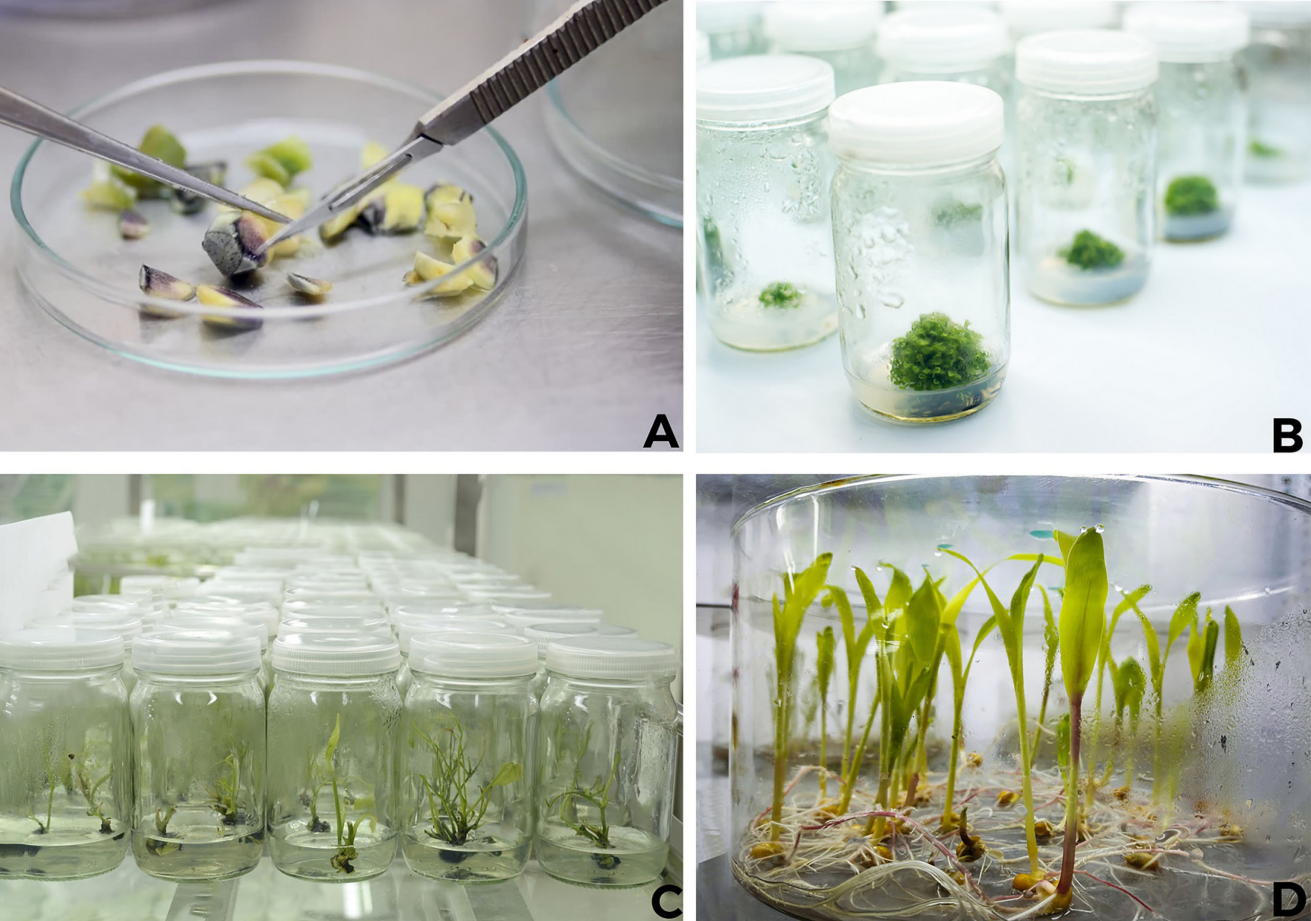
Wheat tissue culture **(A)** Inoculation of single cell to tissue culture media **(B)** Root and shoot development **(C, D)** Roots are fully developed prior to moving plants to pots of soil.

Wheat (*Triticum aestivum* L.) belonging to family poaceae is one of the most important staple crops worldwide as it plays a crucial role in meeting human nutritional requirements (Rashid et al., 2012; Contardo-Jara et al., 2018). To fulfill daily nutritional requirements of growing human population, wheat production should be increased to an annual rate of 2% (Sparks et al., 2014). Wheat tissue culture is one of the alternatives of conventional breeding strategies that may lead to the development of wheat varieties resistant against different diseases (Sparks and Jones, 2014; Hasnain et al., 2018; Hasnain et al., 2020). The transgenic wheat lines conferring resistance against take-all disease which is damaging to plant roots (Liu et al., 2013) and sharp eyespot (Chen et al., 2008) have been developed using tissue culture and biotechnological approaches. Callus culture is widely being used to develop plants conferring resistance against various diseases responsible for drop in crop yield (Efferth, 2019). Over the last few decades, one of the main research objectives is to enhance wheat production in order to meet the demands of increasing human population. The use of different biotechnological approaches is quite promising in attaining these objectives (Contardo-Jara et al., 2018; Hasnain et al., 2018). The transcription factors involved in carbon and nitrogen metabolism pathways are over expressed in wheat varieties, using tissue culture technique, which proved to be promising in increasing wheat yield (Pena et al., 2017; Hasnain et al., 2020) (**Figure 6**).

#### 6.1.4 Rice tissue culture

**Figure 7:**
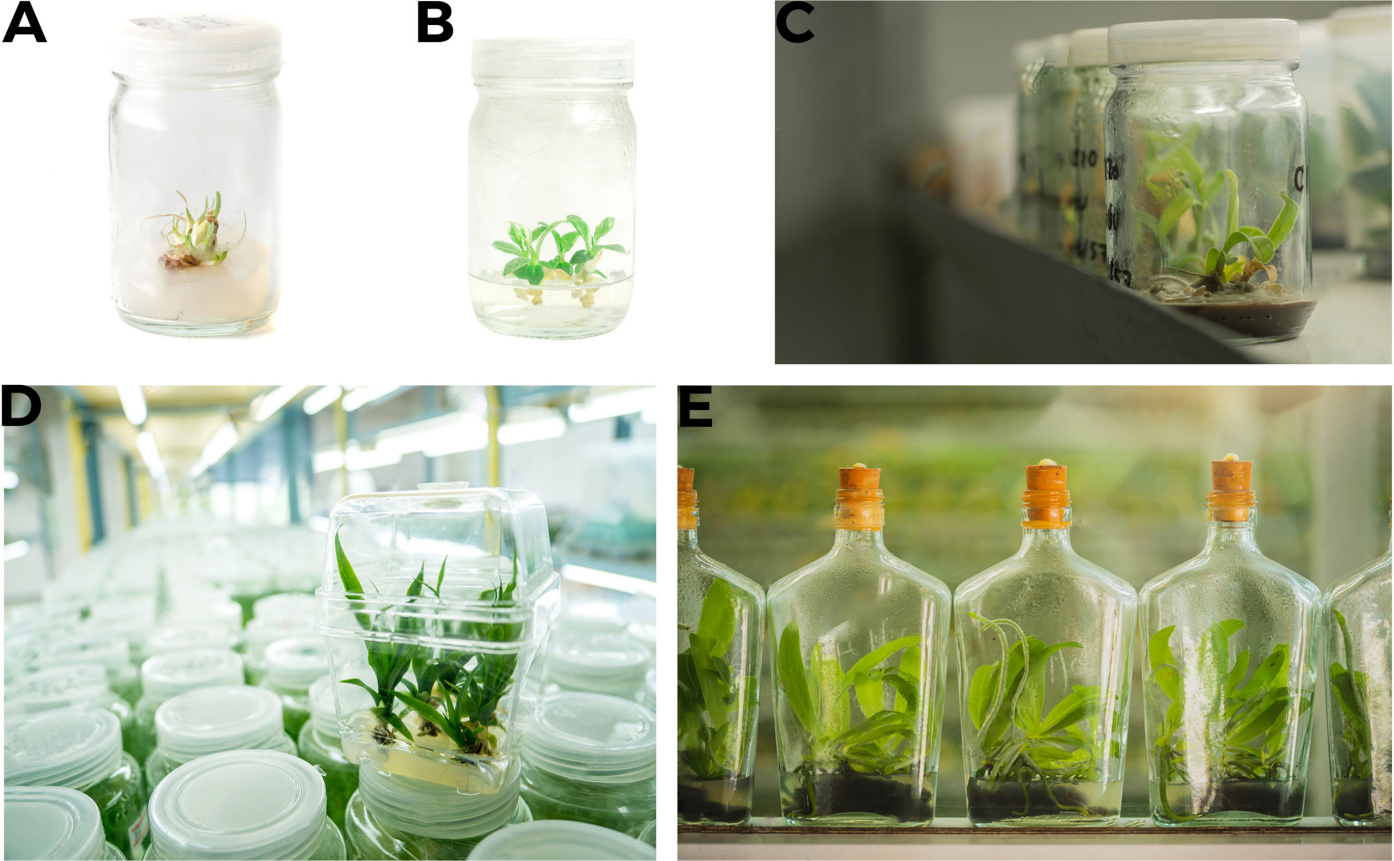
Rice tissue culture **(A–C)** Single cell to callus formation **(D, E)** Sub culturing and regenerated plants are ready to move to pots.

Rice (*Oryza sativa* L.) has been cultivated for more than 7000 years as a major food crop and presently more than 50% of world’s human population is dependent on it (Afolabi et al., 2008; Ahmad et al., 2013). Rice is a rich source of nutritional carbohydrates supplying 50-80% of daily calorie requirements of human population (Jan et al., 2001; Afrasiab and Jafar, 2011). Rice breeders have mostly focused on utilizing natural diversity using hybridization and recombination methods. Some of the rice cultivars including Taichung Native-I, IR-8, Mashuri, IR-36, and other products of are well-known around the world for their better performance against stresses. Despite this, breeders’ attention is occasionally drawn to technologies that provide faster solutions to a wide variety of challenges (Raina, 1989). Among these advancements, plant tissue culture techniques have received the most interest since they promise to provide a variety of approaches to crop improvement difficulties.Tissue culture of rice is one of the approaches of increasing rice production *via* efficient plant regeneration method (Yaqoob et al., 2016; Kaya and Karakütük, 2018). Different explants are used to initiate rice calli which include immature embryos, mature embryos, root segments, coleoptiles and leaf bases that play a significant role in attempts to increase rice yield (Seraj et al., 1997; Li et al., 2007; Benlioglu et al., 2015) (**Figure 7**).

### 6.2 Pharmaceutical industry

Plants are used to treat and prevent particular illnesses and diseases in human beings since the time immemorial (Chandran et al., 2020). Egyptians, Romans, and Chinese give strong proofs of the usage of medicinal plants for the treatment different human ailments (Stafford, 1991; Cowan, 1999). Plants which possess healing metabolites with useful pharmacological effects are referred to as medicinal plants (Atanasov et al., 2015). They are rich in phytochemicals which have the spectacular capability to treat diseases and may be used for the industrial production of pharmaceuticals and nutraceuticals (Chandran et al., 2020; Pant et al., 2021). The medicinal properties of this flora are due to the heterogeneous group of herbal metabolic merchandise called secondary metabolites which can be divergent of their structure and metabolic pathways (Shasmita and Naik, 2018). The extensive research on plant cell culture has caused a surge in the use of this technique in the pharmaceutical industry (Czyz et al., 2014; Hayden et al., 2014; Mali and Chavan, 2016). The manufacturing of pharmaceuticals using culture systems of plants can provide remarkable benefits including cost reduction, quick production, and scalability (Hesami et al., 2017; Espinosa-Leal et al., 2018). Plants are abundant sources of pharmaceutically significant compounds; however, there is a need to manufacture these compounds within stringent laboratory conditions (**Table 2**).

**Table 2:** Plant-derived pharmaceuticals and their uses (Espinosa-Leal et al., 2018).

Plant-Made Pharmaceutical	Plant	Uses	Manufacturer/notes
ELELYSO™ (taliglucerasealfa)	Carrot or tobacco cell culture	Enzyme replacement	Protalix, Carmiel, Israel and Pfizer, USA, ProCellEx^®^ Stable Expression, First plant-made human recombinant therapeutic protein approved (2014)
Vaccine (NDV)	Tobacco suspension cultures	Against Newcastle disease virus	Dow Agrosciences, LLC, Indianapolis, USA, First tobacco cell-based vaccine approved by the FDA against Newcastle disease virus in poultry
VEN150	Rice seeds	For HIV-associated chronic inflammation	Ventria Bioscience, Junction City, KS, USA, Express Tec Stable Expression Scale Cost
Moss-GAA	Moss	Pompe disease	Greenovation Biotech GmbH, Heilbronn, Germany Moss *Physcomitrella patens*-based Broytechnology Speed Scale and Customized
Moss-GBA	Moss *Nicotianabenthamiana* Alfalfa	Gaucher’s disease; Fabry disease; Influenza, Rabies Rotavirus	Greenovation Biotech GmbH, Heilbronn, Germany, Moss *Physcomitrella patens* based Broytechnology, Speed Scale and Customized Medicago, Québec, QC, Canada Proficia™ Transient Expression; Stable Expression
Moss-AGAL			
Vaccines			
Antibody	Duckweed leafy biomass	For non-Hodgkin’s lymphoma	Synthon, Nijmegen, The Netherlands, LEX system Stable expression, Speed quality
Antibody	Tobacco leaves	HIV	Fraunhofer IME, Aachen, Germany, Stable Nuclear Expression Scale Cost
Serum albumin	Rice seed		Healthgen, Wuhan, Hubei, China, Stable Expression, Quality Scale
CaroRx	Tobacco leaves	Dental caries	PlanetBiotechnology, Hayward, CA, USA, Stable Expression Quality Scale
PBI-220	Tobacco leaves	Antibody for anthrax	PlanetBiotechnology, Hayward, CA, USA, Stable Expression Quality Scale
DPP4-Fc		Coronavirus infection	

#### 6.2.1 Secondary metabolite production from plant cultures

The tissue culture experiments on medicinal plants conducted by Veeresham and Chitti (2013) revealed that various secondary metabolites having medicinal values can be obtained from plant cell culture (Bhattacharyya et al., 2017; Shasmita and Naik, 2018). Biotechnological approaches associated with plant tissue culture have increased the scope of medicinal plants along with traditional agriculture used for the industrial production of bioactive metabolites (Matsuura et al., 2018; Pant et al., 2021). Micro-propagation is a valuable technology since many secondary plant metabolites cannot be manufactured chemically (Caldentey and Inze, 2004; Bhattacharyya and Van-Staden, 2016).

#### 6.2.2 Plant cell suspension cultures

An extensive research is being carried out on exploring the biosynthetic properties of plant cell cultures over the last decade (Siahsar et al, 2011). Cell suspension culture methods are currently being employed for large-scale plant cell culture from which secondary metabolites are extracted. A suspension culture is created by moving the comparatively friable component of the callus into liquid media and maintaining it under appropriate physical conditions of aeration, agitation, light, temperature, and other physical factors (Chattopadhyay et al., 2002; Rao and Ravishankar, 2002). Cell cultures not only produce defined standard phytochemicals in huge quantities, but they also reduce the presence of interfering substances found in field-grown plants. The primary benefit of cell cultures is the production of bioactive secondary metabolites in a controlled environment that is independent of climate and soil conditions (Chattopadhyay et al., 2002).

#### 6.2.3 Hairy root cultures

In the last two decades, this technique based on *Agrobacterium rhizogenes* inoculation has gained popularity as a means of creating secondary metabolites generated in plant roots (Palazon et al., 1998). Organized root cultures can contribute significantly to the generation of secondary metabolites. Hairy root disease is caused by *Agrobacterium rhizogenes* in plants. The neoplastic (malignant) roots, generated at rapid growth rate, by *A. rhizogenes* infection are genetically stable and are developed in hormone-free conditions (Hu and Du, 2006). Hairy roots high stability (Giri and Narasu, 2000) and productivity allow them to be used as a powerful instrument for the recovery of important secondary metabolites (Pistelli et al., 2010). Generally, substances released by roots serve as a stockpile of defense chemicals that plants can use to strengthen their inherent defense mechanisms or defend themselves against pathogenic attacks. In order to improve and optimize the number and quality of medicinal plant metabolites, many plant tissue culture techniques have been extensively investigated (Pant, 2014). The following are some of the most often utilized plant-derived metabolites as medicines.

#### 6.2.4 Pharmacologically important plant secondary metabolites

The tissue culture experiments on medicinal plants conducted by Veeresham and Chitti (2013) revealed that various secondary metabolites having medicinal values can be obtained from plant cell culture (Bhattacharyya et al., 2017; Shasmita and Naik, 2018). Biotechnological approaches associated with plant tissue culture have increased the scope of medicinal plants along with traditional agriculture used for the industrial production of bioactive metabolites (Matsuura et al., 2018; Pant et al., 2021). Micro-propagation is a valuable technology since many secondary plant metabolites cannot be manufactured chemically (Caldentey and Inze, 2004; Bhattacharyya and Van Staden, 2016).

Tissue culture has been extensively utilized in breeding programs for over 50 years. The hybrids of such crosses are often sterile due to embryo abortion but can be ‘rescued’ by means of culturing or transplanting the embryos. The medium utilized for cell culture can be optimized for the production of desirable products (Atanasov et al., 2015; Pant et al., 2021). Various plant tissue culture systems have been extensively studied to improve and enhance the production and quality of plant metabolites produced by the medicinal plants (Pant, 2014).

Alkaloids are the structurally diverse group of secondary metabolites which possess significant biological activities (Moreira et al., 2018). Plants make them as a defense mechanism in response to biotic and abiotic stressors (Taha et al., 2009), hence, several plants produce them in reaction to caterpillars that feed on them, and they are potentially dangerous (Hussain et al., 2018; Peng et al., 2019). The invasion species’ parasympathetic nervous system is blocked by these alkaloids. Plant tissues would collect very little or no alkaloids if this sort of aggression did not exist (Apone et al, 2010). The following are some of the most commonly utilized alkaloids in the pharmaceutical business:

The isoquinoline alkaloid berberine (BBR) is extracted from the roots, stems, and rhizomes of Coptis chinensis, Coptis japonica, and various other herbal plants and used in oriental medication for hundreds of years **(** Peng et al., 2019 **)**. Acupuncture, Chinese herbal medicines, oriental nutrition, and dietary therapy, and tuina or oriental bodywork are the five major branches of oriental medicine, which are the oldest codified system of medicine (Chen et al., 2017; Yu et al., 2018). They restore health and balance by treating illness through five major branches of oriental medicine, which include acupuncture, Chinese herbal medicines, oriental nutrition and dietary therapy, and tuina or oriental bodywork. Plant extracts are used to make Oriental remedies (berberine chloride) (Zhang et al., 2017). Moreover, berberine is typically used as an oral medicine for the treatment of excessive cholesterol, high blood pressure, diabetes, or excessive levels of lipids in the blood (Kawano et al., 2015; Jing et al., 2018). It is also used as a topical remedy for canker sores and burns (Cui et al., 2018).

**Figure 8:**
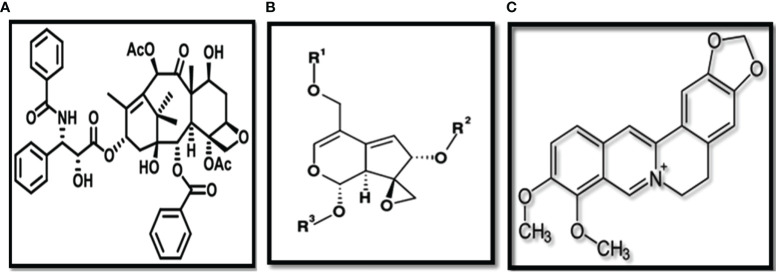
Structures of **(A)** Berberine **(B)** Valepotriates **(C)** Taxol.

Valepotriates is the name given to a set of compounds determined to have tranquilizing results, in addition to proof of having antitumor and cytotoxicity effects (Boss et al., 2002; Patocka and Jakl, 2010). *Valerianaceae*, consisting of herbs and rarely shrubs, has been used for decades to obtain medicinal drugs. Traditionally, valepotriates has been used to treat spastic colitis and gastrointestinal pain (Becker and Chavadej, 1988; Andreatini and Leite, 1994). The tissues of *Valerianaceae*,placed in a growth medium, are used to investigate which species produced better levels of the desired compounds and are resistant against various stresses. A lot more research is being done on the micro-propagation of *Valerianaceae* to explore the compounds with medicinal value (Boss et al., 2002; Jarema, 2008) (**Figure 8**).

Another compound is Taxol which was generically known as paclitaxel, was granted FDA (Food and Drug administration) approval in 1992 (Khosroshahi et al., 2006). The drug is administered to ovarian cancer patients and is often used as a secondary treatment when chemotherapy is failed in these patients (Badi et al., 2015). Taxol is mainly extracted from the bark of wild *Taxus brevifolia* trees, however, due to its increased demanda as an anti-cancerous drug; researchers are primarily interested in its *in vitro* propagation of taxol producing flora for a more rapid and environment friendly technique (Walker et al., 2004; Nasr et al., 2007). As a result, plant tissue culture is a viable technology for producing desirable bioactive chemicals from plants. Plant tissue culture is also used to help save endangered species, as many therapeutic plants are on the verge of extinction due to overuse (Rout et al., 2000; Bestoso et al., 2006; Chen et al., 2016c; Vidyagina et al., 2021). Further investigations on various plant species having medicinal values is needed to explore as most of the natural flora is still unidentified. The development of plant tissue culture techniques will expand the long-term use of therapeutic plants in the future (Balunas and Kinghorn, 2005).

### 6.3 Cosmetic Industry

Over the last ten years, the enticing trend of natural cosmetics production has ushered in a new era of plant cell culture technology and during this time, more than 50 cosmetic products based on extracts of plant cell cultures have been developed, the bulk of which are made with plant cell suspension cultures (Charles et al., 2017; Krasteva et al., 2020). Plant cell culture cosmetic production is not dependent on appropriate seasonal conditions; thus, it requires less time and energy. Cosmetic extracts derived from plant cell cultures suit the market’s increasingly stringent safety requirements. In addition to being free of pathogens, pollutants, and pesticide residues, plant cells generated under aseptic laboratory conditions rarely include any malignant compound or potential allergen, which would otherwise destroy the majority of the plant extracts obtained (Schmid et al., 2008; Trehan et al., 2017; Eibl et al., 2018). Plant cell suspension cultures are cultivated in single-use wave-mixed bioreactors or renewable stainless steel stirred bioreactors in commercial production today. Single-use wave-mixed bioreactors are perfect for personalized goods due to their modest operational capacity, improved security, and more quick and simple operation (Barbulova et al., 2014; Krol et al., 2020).

In recent decades, due to increased customer demand for cutting-edge cosmetic formulations made with efficient, secure, and sustainable components, the cosmetic industry has expanded internationally and become extremely competitive (Zappelli et al., 2016). Plant tissue cultures are a perfect source of safe and pure components for cosmetic goods since they can be cultivated under controlled conditions with minimum possibility of pathogen or environmental contamination. Utilizing various extraction techniques and solvents while taking advantage of the chemical makeup of plant cell components, plant tissue culture technology allows the isolation of many active ingredients from a single culture (Apone et al., 2018). Discussed below are *in vitro* propagated plant species specifically known for producing valuable pyhtocompounds with potential uses in cosmetics: (**Table 3**).

#### 6.3.1 Bean (Dolichos biflorus)

A hydrosoluble extract of *Dolichos biflorus* cell cultures was examined for the amount of isoflavones such genestin and daidzen, and their glucosidic derivatives. In addition to avoiding cellular damage, these substances also reduce the inflammation caused by ultraviolet (UV) light in dermal and epidermal cells, known as solar erythema (sunburn), which is characterized by skin reddening brought on by an increase in blood flow and capillary dilation (Bimonte et al., 2014).

#### 6.3.2 Shrub (*Daphne odora*)

Another illustration of the characterization of a plant cell culture extract for cosmetic uses comes from the evergreen shrub *Daphne odora*, which is particularly interesting because it can withstand low temperatures. The ability of a hydrosoluble extract from shrub cell cultures to alleviate cutaneous irritation by cold stress was investigated (Bimonte et al., 2018).

#### 6.3.3 Raspberry *(Rubus ideaus*)

The expression of genes related to skin hydration, including aquaporin 3, filaggrin, and involucrin, was induced by the liposoluble extract made from raspberry cell cultures. Additionally, the extract boosted the expression of the ceramide-producing enzymes glucocerebrosidase and hyaluronic acid synthase and stimulated their activity. Additionally, it demonstrated an extraordinary capacity to hydrate skin when tested on human skin *in vivo*, indicating that it had tremendous potential as a skin care agent, particularly for dry and ageing skin (Titos et al., 2015).

#### 6.3.4 Apple (Malus domesticus)

The hydrosoluble and liposoluble fractions of *Malus domesticus* (apple) cell suspension cultures were combined, the entire cell lysate was subjected to high-pressure homogenization, and they were then added to the finely dispersed liposomes to create a cosmetic ingredient (nanosomes). The expression of the antioxidant enzyme Heme Oxigenase 1 was first examined to gauge the preparation’s anti-senescence effects. Four weeks of treatment resulted in a 16% reduction in wrinkle depth, according to clinical studies (Schmid et al., 2008). Additionally, it has been demonstrated that this extract is excellent at shielding human stem cells from UV rays.

#### 6.3.5 Rose mallow *(Hisbiscus syriacus*)

Another example of an active ingredient created for use in skin care products comes from rose cell suspension cultures (Di Martino et al., 2017). Flavonoids and coumarins, which are known for having tissue-regenerating capabilities, were found in the hydroethanolic extract of *H. syriacus* cellsin a chemical study (Borges Bubols et al., 2013). On both fibroblasts and keratinocytes, the extract was examined for its capacity to hydrate wounds and promote wound healing. Ex vivo studies of human skin biopsies from injured people confirmed these findings in which topical applications of *H. syriacus* extract dramatically improved wound closure by increasing the production of neo-epidermis (Di Martino et al., 2017)

#### 6.3.6 Butterfly bush *(Buddleja davidii)*

In plant cell suspension cultures of the butterfly bush *(Buddleja davidii*), the chemical verbascoside, a phenylpropanoid glycoside, renowned for its antioxidant, anti-inflammatory, photoprotective, and chelating activities was generated in high concentrations (Vertuani et al., 2011). *In vivo* investigations showed that this substance suppressed the activity of collagenases linked to skin ageing as well as the activation of pro-inflammatory factors.

#### 6.3.7 Coffee (Coffea bengalensis)

Bengal coffee *(Coffea bengalensis*) plant cell culture extract was created as an active ingredient to support skin health and homeostasis because it did not include the alkaloid caffeine. Caffeine’s anti-obesity properties increase its utility in cosmetics. However,it has been investigated that oral use of caffeinedecreased the thickness of hypodermis, which is linked to wrinkle formation.

#### 6.3.8 Trefoil (*Lotus japonicas*)

In a recent study, a peptide/sugar mixture that was extracted from the cell walls of cultures enriched with somatic embryos of *Lotus japonicus* plant was considered as a possible skin-rejuvenating element (Tito et al., 2019). When the combination was chemically characterized, it was discovered to include a significant sugar fraction with high concentrations of glucose, galactose, mannose, and fructose. Saccharides are recognized to have advantageous effects on hydration with anti-inflammatory effect on dermal cells. As a matter of fact, the function of sugars in cosmetics has not been fully understood, more research needs to be done to explore the underlying mechanism (Tolg et al., 2014 **).**

Plant cell cultures are now being used for the production of ‘cosmeceuticals’, products having cosmetic as well as therapeutic (medical or drug-like) effects that exert beneficial effects on skin health. An extensive investigation is being done to investigate the plant sources producing active ingredients, such as antioxidants, ingredients with antimicrobial, anti-viral, anti-cancerous, anti-fungal, anti‐inflammatory, and anti-allergy properties along with moisturizing, anti‐ageing, anti‐wrinkle and UV protective properties, which are crucial to cosmetics industry  (Apone et al., 2010; Tito et al., 2011; Morus et al., 2014). Most of the phytochemicals such as polyphenols, phenolics acids, triterpenes, flavonoids, stilbenes, steroids, carotenoids, steroidal saponins, sterols, fatty acids, polysaccharides, sugars, and peptides are extracted with relevant solvents and utilized as active constituents in cosmetic preparations (Barbulova et al., 2014; Furusaki and Takeda, 2017).

Applications of plants/flowers extracts in cosmetics are significant which include skin moisturizing, whitening or tanning products, sunscreens, radical-scavenging antioxidants, immune stimulants, and skin thickeners etc. (Ochoa‐Villarreal et al., 2016; Georgiev et al., 2018). Because of its ability to minimize wrinkles in the crow’s feet area of the face, a liposome-encapsulated extract of cultured apple stem cells is employed as an ideal ingredient in anti-aging products (Satish et al., 2019; Krasteva et al., 2020; Krol et al., 2020). Chemicals isolated from *Catharanthusroseus* are currently used in the manufacturing of both ordinary consumer and professional care cosmetics, demonstrating significant potential in protecting skin from heavy metal toxicity (Mallurwar and Pathak, 2008; Schmid and Zülli, 2012; Blum et al., 2013; Hayta et al., 2017; Meir et al., 2018). Moreover, the supplements that lessen hair loss and skin aging by nourishing skin and boosting body’s immune system are being produced *via* plant tissue culture (Ribeiro et al., 2015; Murthy et al., 2019). The concentrates from unique or endangered plant species can also be made accessible for cosmetic production using plant cell culture techniques (Blum et al., 2013; Ribeiro et al., 2015; Chen et al., 2016b; Murthy et al., 2019). Melanin is one of the most extensively dispersed pigments found in bacteria, fungi, and animals, therefore, suppression of the melanin-producing enzyme tyrosinase is one of the most frequent defense strategies used by the roots (Apone et al, 2018). Human skin color is largely influenced by the amount of melanin produced by melanocytes, and tyrosinase controls this pigment manufacture. Thus, one of the primary objectives of skin lightening substances in cosmetics is to reduce the effects of sun exposure and age spots, which are mostly constituted of melanin and give the skin an even tone. Sena et al. (2018) examined the depigmentation effects of two different extracts made from the hairy root cultures of, *subsp. pekinensis* (Chinese cabbage) and *Brassica Rapa*. Some popular plant-derived active cosmetic ingredients that are currently available in the market as mentioned and elaborated in **Table 3**.

**Table 3:** Popular active cosmetic ingredients derived through plant cell culture technology (Krasteva et al., 2020).

Product	Plant Source	Active compound(s)	Application(s)	Manufacturer	Web page
PhytoCellTec™ Symphytum	*Symphytum officinale* L.	Water extract	Boosts the regenerative power of epidermal stem cells, increases epidermal thickness, improves barrier function	Mibelle AG Biochemistry	https://mibellebiochemistry.com/phytocelltectm-symphytum
PhytoCellTec™ nunatak^®^	*Saponaria pumila* L.	Water extract	Maintains dermal stem cell vitality after UV irradiation, improves skin density, elasticity and firmness	Mibelle AG Biochemistry	https://mibellebiochemistry.com/phytocelltectm-nunatakr
Deobiome Noni	*Morinda citrifolia* L.	Anti‐quorum sensing molecules; sugars	Protection and balance of microbiome, Metabolism shift from lipids to polysaccharides, Quorum-sensing inhibitors, Skin health and detoxification	Vytrus Biotech	https://www.vytrus.com/products/deobiome-noniprcf-the-biological-deodorant/?portfolioCats=17
Arabian Cotton PRCF	*Gossypium herbaceum* L.	Phenols and flavonoids	Anti‐oxidants, Photoprotective (sun-block), enhances cell viability, modulates inflammatory response in human epidermal progenitor cells	Vytrus Biotech	https://www.vytrus.com/products/arabian-cottonprcf-the-broad-spectrum-protector-against-photo-aging/?portfolioCats=17

## 7 Limitations

Despite of various useful applications of plant-derived compounds obtained *via in vitro* plant propagation, there are certain limitations which need to be addressed in order to reap maximum benefit of this technique. The limitations include difficulties with continuous operation, product removal, and aseptic conditions. A few culture systems appear to have the potential to become commercially viable because of these limitations (Goel et al., 2011). Furthermore, the prevalence of somaclonal variation in populations formed from tissue culture has a detrimental impact on the utilization of tissue culture and has remained a serious concern. Somaclonal variation is variance that originates in cell and tissue cultures. At the moment, the word somaclonal variation refers to all types of tissue culture produced variations (Bajaj, 1990), Since Braun (1959) first observation and description of somaclonal variation; it has been one of the key issues of many tissue cultivated plants. Plant cell development *in vitro* and regeneration into full plants is an asexual process that involves just mitotic division of the cell and, ideally, should not result in variation. Clonal multiplication of genetically homogenous plants is the ideal scenario. Uncontrolled and unpredictable spontaneous variation throughout the cultural process is thus an unanticipated and largely undesirable phenomenan (Ranghoo-Sanmukhiya, 2021). This is attributed to somaclonal variation in production clones and low secondary metabolite titers (Sharma et al., 2014). In contrast to these detrimental consequences, its use in crop improvement through the development of new variations is widely recognized.

The expense of culture material, electricity, and labor are other issues with *in vitro* tissue cultivation. Alternative materials such as home sugar or other sugars as carbon sources, as well as various types of starches and plant gums in place of agar, have been used in numerous experiments to overcome this problem. Alternatives have included liquid media and cell suspension cultures, temporal immersion systems, and reusable glass beads as support matrices (Etienne and Berthouly, 2002; Ahloowalia and Savangikar, 2003; Sahu and Sahu, 2013; George and Manuel, 2013). Bioreactors and robotic propagule handling, for example, have been shown to save production costs. Another issue that occurs with plant tissue culture is the plants’ genetic stability. Somaclonal differences that occur during *in vitro* propagation, commercial phytochemical synthesis, or genetically modified plants can have significant economic consequences and are a major impediment to the practical application of plant tissue culture techniques for the production of active metabolites (Rahman and Rajora, 2001). As a result, the genetic constitution and stability of *in vitro*-regenerated plants must be monitored and examined in order to screen somaclonal variability within a cell culture. As part of the techniques utilized in that process, several strategies are used to analyze possible adjustments at various levels (Bhattacharyya and Van-Staden, 2016).

## 8 Conclusion and future perspectives

Plant micro propagation is a powerful technique in order to acquire plant extracts with various commercial applications than using whole plants. The application of targeted genome engineering, notably the previously stated genome editing mediated by CRISPR/Cas9, is one of the most important techniques. Using this technical approach, new plant kinds can be created without the input of foreign genes (Doudna and Charpentier 2014; Baltes and Voytas 2015; Nogueira et al., 2018). Furthermore, the cosmetics sector which introduces hundreds of new cosmetics items every year is heavily influenced by customer demand. Plant cell culture extracts with several particular actions for skin care, make-up, and hair care as supplement components are gaining popularity in the cosmetics sector. Consumers desired cosmetics that are effective, safe and natural can be obtained by exploring phytochemicals with these desired properties under *in vitro* conditions. The development of efficient and appealing novel active ingredients for cosmetics, and skin care in particular, is developing in this direction. In this regard, plant tissue culture methods hold enormous promise. In the future, plant tissue cultures might not only be a source of novel chemicals with uncharted biological actions, but they might also work as alternative recombinant protein biofactories, particularly for those whose expression might be problematic or constrained in fermenting microbes. In the next decade, tissue culture technique should attain its full potential, thanks to new technologies like gene editing and environmental component manipulation. However, the major constraints need to be addressed for sustainable industrial applications of *in-vitro* regenerated plants on a large scale. The tissue culture experiments on medicinal plants conducted by Veeresham and Chitti (2013) revealed that various secondary metabolites having medicinal values can be obtained from plant cell culture (Bhattacharyya et al., 2017; Shasmita and Naik, 2018). Biotechnological approaches associated with plant tissue culture have increased the scope of medicinal plants along with traditional agriculture used for the industrial production of bioactive metabolites (Matsuura et al., 2018; Pant et al., 2021). Micro-propagation is a valuable technology since many secondary plant metabolites cannot be manufactured chemically (Caldentey and Inze, 2004; Bhattacharyya and Van Staden, 2016).

## Author contributions

AH, SA, FK, ME, SN, AA and AB designed and wrote the manuscript. MH, GM and SI have done graphical work. AB, RA, DM and MM helped to revise the manuscript. AH, SN, SI, AB, MH and MA critically revised and supervised the manuscript. RA and DM provided the funds for the publication of this review article. All authors contributed to the article and approved the submitted version.

## Acknowledgments

We thank the Faculty of Process and Environmental Engineering Łódź University of Technology, Poland for providing financial support to the current study.

## Conflict of interest

The authors declare that the research was conducted in the absence of any commercial or financial relationships that could be construed as a potential conflict of interest.
