# Corrigendum: Plants *in vitro* propagation with its applications in food, pharmaceuticals and cosmetic industries; current scenario and future approaches

**DOI:** 10.3389/fpls.2023.1197747

**Published:** 2023-04-17

**Authors:** Ammarah Hasnain, Syed Atif Hasan Naqvi, Syeda Iqra Ayesha, Fatima Khalid, Manahil Ellahi, Shehzad Iqbal, Muhammad Zeeshan Hassan, Aqleem Abbas, Robert Adamski, Dorota Markowska, Alaa Baazeem, Ghulam Mustafa, Mahmoud Moustafa, Mohamed E. Hasan, Mohamed M. A. Abdelhamid

**Affiliations:** ^1^ Institute of Molecular Biology and Biotechnology, The University of Lahore, Lahore, Pakistan; ^2^ Department of Plant Pathology, Faculty of Agricultural Sciences and Technology (FAST), Bahauddin Zakariya University, Multan, Pakistan; ^3^ College of Plant Sciences and Technology, Huazhong Agricultural University, Wuhan, China; ^4^ State Key Laboratory of Agricultural Microbiology and Provincial Key Lab of Plant Pathology, Huazhong Agricultural University, Wuhan, China; ^5^ Faculty of Process and Environmental Engineering, Lodz University of Technology, Lodz, Poland; ^6^ Department of Biology, College of Science, Taif University, Taif, Saudi Arabia; ^7^ Department of Agriculture (Extension and Adoptive Research), Agriculture Extension Department of Government of Punjab, Lahore, Pakistan; ^8^ Department of Biology, Faculty of Science, King Khalid University, Abha, Saudi Arabia; ^9^ Department of Botany and Microbiology, Faculty of Science, South Valley University, Qena, Egypt; ^10^ Bioinformatics Department, Genetic Engineering and Biotechnology Research Institute, University of Sadat City, Sadat City, Egypt; ^11^ Agricultural Botany Department, Faculty of Agriculture (Saba Basha), Alexandria University, Alexandria, Egypt

**Keywords:** plant tissue culture, explants, secondary metabolites, industry, pharmaceuticals, medicines, cosmetics

In the published article, there was an error in **Figure 3** as published. The diagram in **Figure 3** of our published manuscript is very similar to that of a previously published article. The corrected **Figure 3** and its caption appear below.

**Figure 3:**
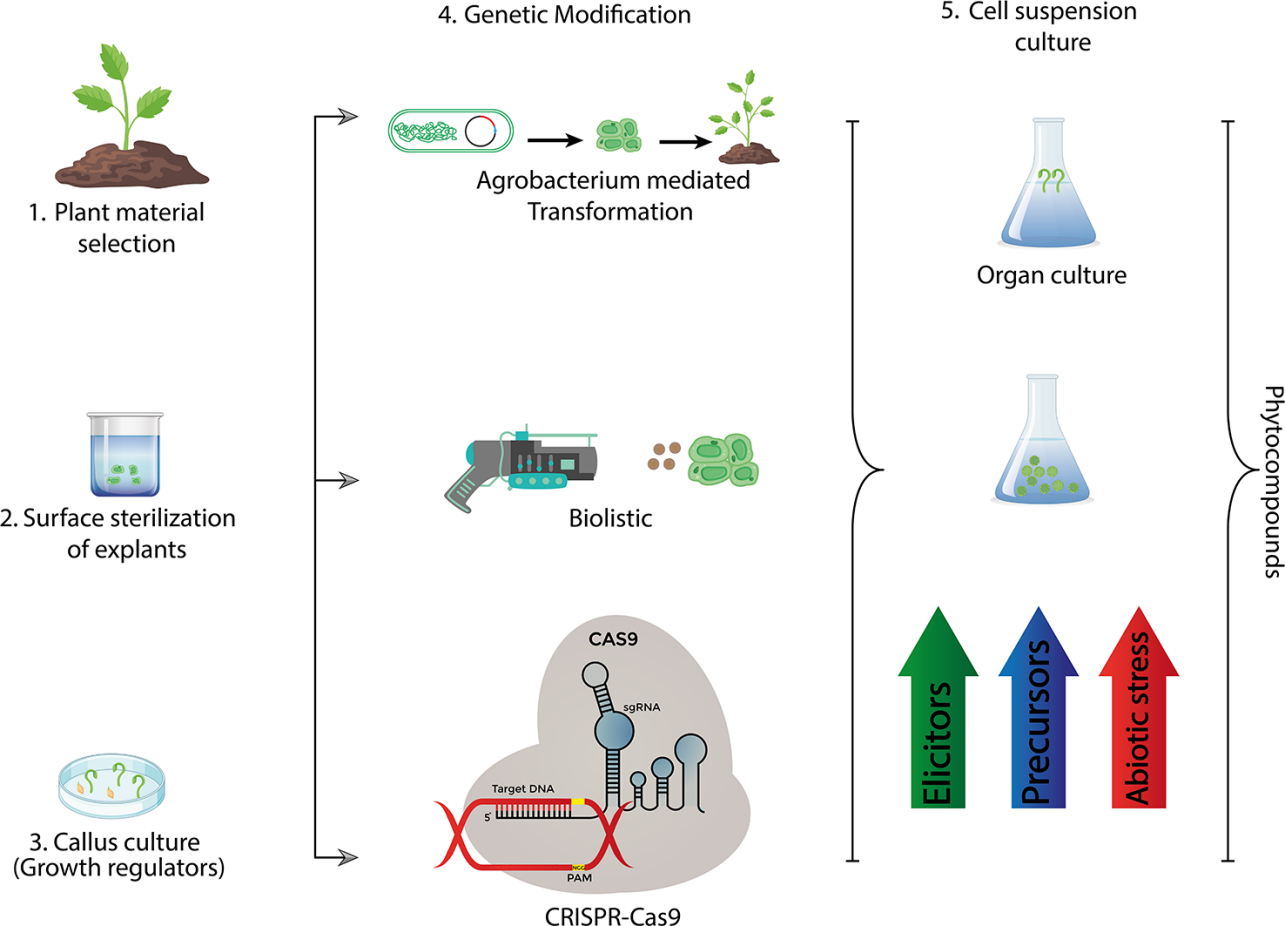
Recent methods used for industrial production of bioactive compounds via plant tissue culture.

The authors apologize for this error and state that this does not change the scientific conclusions of the article in any way. The original article has been updated.

